# Digital manufacturing of personalised footwear with embedded sensors

**DOI:** 10.1038/s41598-023-29261-0

**Published:** 2023-02-03

**Authors:** Marco R. Binelli, Ryan van Dommelen, Yannick Nagel, Jaemin Kim, Rubaiyet I. Haque, Fergal B. Coulter, Gilberto Siqueira, André R. Studart, Danick Briand

**Affiliations:** 1 Complex Materials, Department of Materials, ETH Zürich, 8093 Zürich, Switzerland; 2 Soft Transducers Laboratory, EPFL Lausanne, 2000 Neuchâtel, Switzerland; 3 Cellulose & Wood Materials Laboratory, Empa, 8600 Dübendorf, Switzerland

**Keywords:** Electronic devices, Design, synthesis and processing, Soft materials

## Abstract

The strong clinical demand for more accurate and personalized health monitoring technologies has called for the development of additively manufactured wearable devices. While the materials palette for additive manufacturing continues to expand, the integration of materials, designs and digital fabrication methods in a unified workflow remains challenging. In this work, a 3D printing platform is proposed for the integrated fabrication of silicone-based soft wearables with embedded piezoresistive sensors. Silicone-based inks containing cellulose nanocrystals and/or carbon black fillers were thoroughly designed and used for the direct ink writing of a shoe insole demonstrator with encapsulated sensors capable of measuring both normal and shear forces. By fine-tuning the material properties to the expected plantar pressures, the patient-customized shoe insole was fully 3D printed at room temperature to measure in-situ gait forces during physical activity. Moreover, the digitized approach allows for rapid adaptation of the sensor layout to meet specific user needs and thereby fabricate improved insoles in multiple quick iterations. The developed materials and workflow enable a new generation of fully 3D printed soft electronic devices for health monitoring.

## Introduction

Standards in healthcare are continuously improving as the demand for more accurate and personalized health monitoring continues to grow^1–7^. This demand not only originates from the medical sector, seeking to address strictly clinical needs, but also from athletes and sports enthusiasts who wish to become more aware of their health status and physical condition^3,8^. To address this, personalized soft wearable sensing systems are under development to provide physiological health metrics over extended time^1^ without sacrificing the comfort of the user^9,10^. One target application for continuous health monitoring is the analysis of gait, which can provide insight into overall health^11^, aging^12,13^, sports performance and injury recovery^14^. While many advances have been made in terms of materials and sensors development to realise gait-monitoring wearables^1,9^, few complete solutions exist that can be easily tailored to the user. Furthermore, the gold standard in gait motion measurements continues to rely on stationary instrumentation^10,15^, which cannot be used for free-living monitoring. In this context, inertial sensors have shown some promise as a wearable solution^16^. However, the required measurement protocols to utilise these are still under development and long-term monitoring with patient-specific devices has not yet been demonstrated^17^.

Electronic footwear in the form of socks and insoles with integrated sensors offer an attractive strategy to reliably measure gait^10,18^, while offering a high degree of comfort for the user. Because they can easily and non-intrusively be inserted into a shoe, insoles are ideal candidates for gait motion monitoring. Tailoring the shape, position and material of the insole also provides the opportunity to enhance the gait and prevent further health problems by correcting posture, and improving the plantar pressure distribution^14,19^. Moreover, sports performance can be positively impacted by the use of insoles with tuneable stiffness and geometry^20^. The integration of sensors in state-of-the-art insoles is an open manufacturing challenge, for which different concepts have been proposed. So far, several elastomeric smart plantar sensing systems have been developed with integrated mechanical sensing mechanisms including capacitive^21,22^, piezoresistive^23^, force-sensitive resistor^24^ and triboelectric^25,26^ pressure sensors. Such integrated systems have been manufactured using either reel-to-reel fabrication^27^, laser-induction^28^, or cleanroom fabrication^29^. Despite these enticing developments, current approaches still rely on conventional manufacturing workflows that cannot meet the increasing demand for digitalisation and personalisation.

3D printing is a promising approach to fill this gap by providing high levels of customization, short production cycles and full digitalisation opportunities^30,31^. Even though the sports industry has expressed much interest in personalized 3D printed objects^32^, applied research on the fabrication and characterization of such devices has been lagging behind. Technologies for 3D printing the soft materials necessary for the fabrication of wearable electronics are already available. These include vat photopolymerization^33,34^, material jetting^35^ and material extrusion techniques like direct ink writing (DIW) and fused filament fabrication (FFF)^36–39^. In particular, DIW is suitable due to the myriad of materials that can be deposited using this technique^33,34,40,41^, notably polymers, conductive pastes, as well as piezoresistive and piezoelectric materials^42–49^. In spite of the extensive material palettes available, this versatile 3D printing technique has not yet been fully exploited for the fabrication of electronic footwear. Recent work on DIW of soft electronics has demonstrated the potential of this technology in generating sensor arrays for insole applications^21^. 3D printing of user-specific insoles and their validation in free-living environments are crucial next steps to create the next generation of personalized electronic footwear.

**Figure 1:**
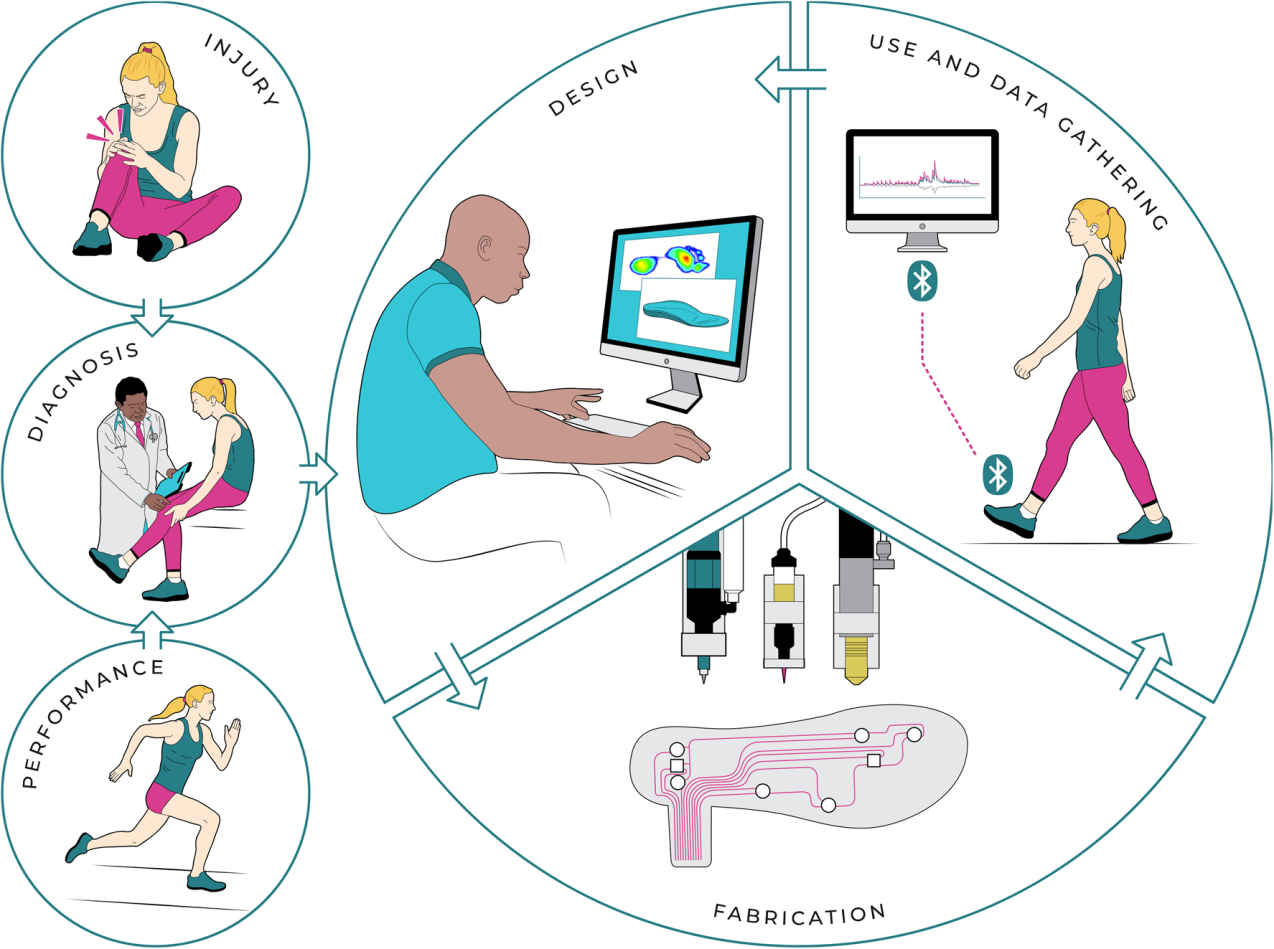
Proposed analysis and fabrication cycle of customized insoles with embedded sensors. In the envisioned workflow, a trained healthcare worker can 3D print the insole based on the initial medical diagnosis and later print adapted versions of it considering the gait data captured during physical activities. Illustration provided by Estevam Quintino (CC BY 4.0).

In this work, we propose an integrated 3D printing platform for the digital manufacturing of a fully customized smart insole with embedded piezoresistive sensors, and demonstrate the use of such personalized footwear in real-world physical activities. Using functional inks that provide both sensing capabilities and local tuning of the insole’s mechanical properties, our aim is to harness the multimaterial, complex shaping capability of the DIW to not only gather data from user–environment interactions but also tune the mechanical response of the personalized insole to improve the performance or health condition of the user (Fig. 1). In the long term, the vision is to utilize this printing platform to acquire real physical data, which can be used as input for the creation of a digital twin to generate enhanced footwear designs. To this end, the mechanical and rheological properties of silicone composites, with functional fillers, are first characterized and optimized to enable the fabrication of complex 3D structures with integrated pressure and shear sensors. Next, we validate the performance of our sensors and insole designs through mechanical tests that simulate static and dynamic loads of a walking person. Finally, we shape an insole with embedded sensors by reconstructing the surface of a commercial shoe insole, and perform tests with the insole inside a shoe to capture gait in free‐living conditions.

## Results and discussion

### Ink formulations

**Figure 2:**
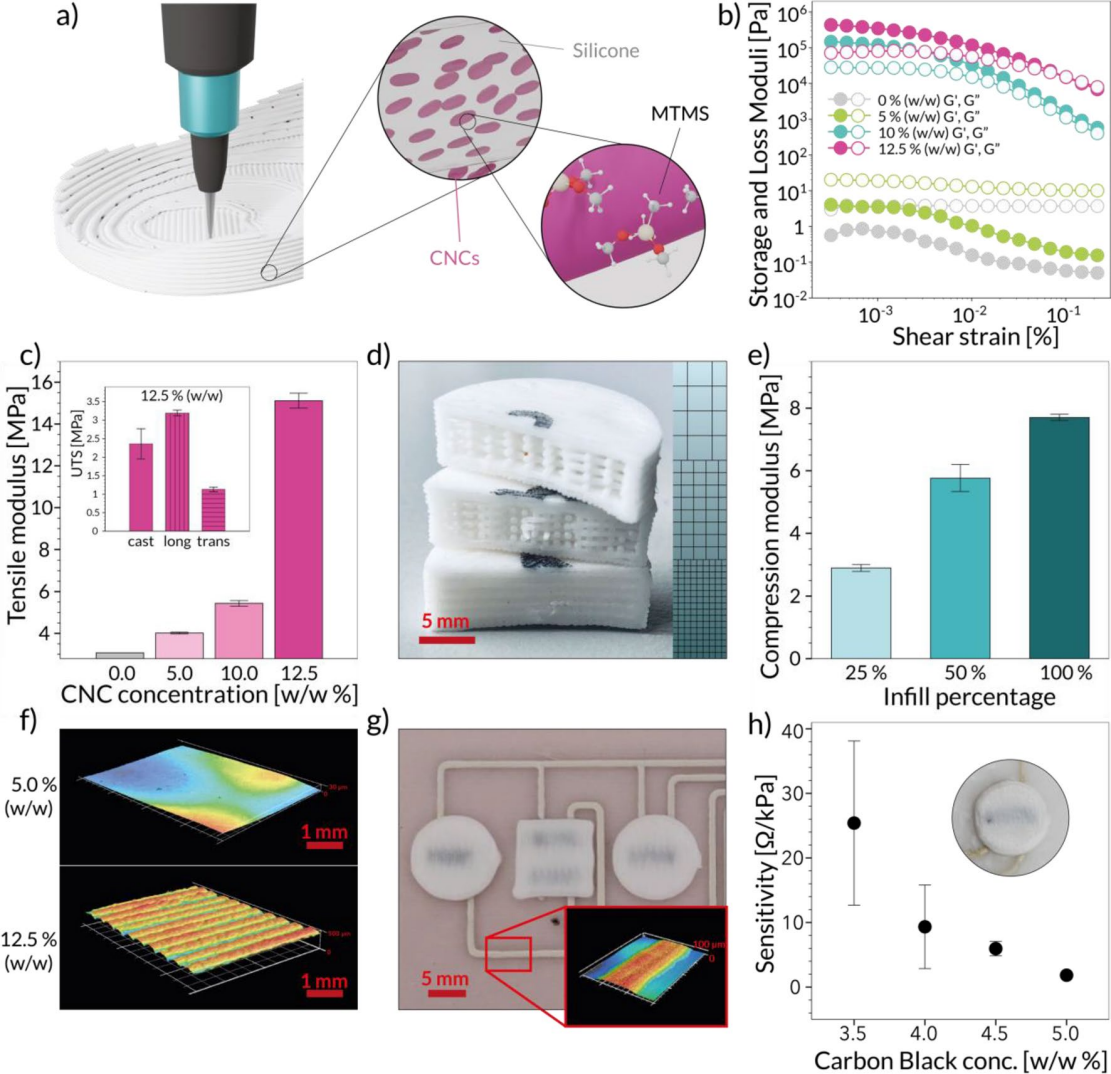
Design and characterization of structural and piezoresistive inks. (**a**) Schematic of the DIW printing process of the CNCs-reinforced silicone resin used as structural ink. The cartoons illustrate the alignment of the surface-modified CNCs within the silicone matrix. (**b**) Storage and loss shear moduli of structural inks with different concentrations of CNCs. (**c**) Tensile moduli of composite inks prepared with distinct CNCs concentrations. The inset displays the effect of the printing direction on the tensile strength of the printed parts. (**d**) Grid-type structures with different infill densities printed with 12.5% (w/w) CNCs-reinforced ink. From bottom to top, the infill densities correspond to 100%, 50%, and 25%. (**e**) Compression moduli of the 12.5% CNCs-reinforced ink at different infill densities. (**f**) Surface 3D reconstructions of structural inks contain 5.0% (w/w) (top) and 12.5% (w/w) (bottom) CNC concentrations. Both samples were printed using a nozzle with a diameter of 0.62 mm. (**g**) Surface 3D reconstruction of a printed silver connector on a substrate printed with a 5.0% CNCs-reinforced ink. (**h**) Effect of different carbon black concentrations on the sensitivity of piezoresistive elements printed from inks containing 1-pentanol as diluent. Inset, sample normal sensor used for the determination of sensitivity.

The proposed digital manufacturing platform relies on the development of a suitable materials palette. To this end, we prepared a set of inks that match the rheological behaviour required for DIW and that also feature the material properties necessary to print functional sensors and mechanically tuneable 3D structures. To match the required flexibility and elasticity for a wearable device, our formulations are based on a commercially available silicone elastomer, which we compounded with two types of functional filler particles. First, surface-modified cellulose nanocrystals (CNCs) were used as structural filler to modify the rheological properties of the inks and the mechanical response of load‐bearing parts of the insole. The nanocellulose is functionalized with methyltrimethoxysilane (MTMS) to enhance its surface affinity towards silicone, and thus facilitate mixing with the base elastomer (Fig. 2 a). Second, functional fillers in the form of carbon black particles were incorporated into rheologically optimized silicones to print the piezoresistive elements of the sensors of the insole. These electrically conductive filler particles are expected to form a percolating network within the silicone-based composite, which makes its electrical resistance change upon the application of external forces.

By incorporating the modified CNCs into the silicone matrix we are able to tune both the rheological properties of the inks and the mechanical response of the printed material after curing (Fig. 2b). In terms of rheological behaviour, the ink changes from a fluid to a viscoelastic material when the CNC concentration is increased beyond 5.0% (w/w). The mechanical properties of the cured ink are also strongly affected by the presence of CNCs. Tensile tests show that the network-forming ability of the CNC particles leads to 2-times higher strength and 5-times higher stiffness when compared to those of pure silicone (Fig. 2c). The addition of CNCs does not only allow us to change the rheological properties and the stiffness of the ink, but also to obtain an anisotropic mechanical response. Due to the shear and extensional forces experienced by the material during extrusion through the nozzle, the elongated CNC particles align in the direction of the applied flow (Fig. 2a), as demonstrated in earlier work^50^. This results in a printed line with anisotropic microstructure and properties (Fig. 2c, inset) and gives us the opportunity to further act on the strength of the printed insole just by determining the moving pattern of the extruder during the printing process. In addition to the ink formulation, the mechanical properties of the printed parts can also be easily tuned by changing the density of print lines in grid-type structures (Fig. 2d). Indeed, an increase in fill factor from 25 to 100% was found to enhance the compressive stiffness of the grid from 3 to nearly 8 MPa (Fig. 2e).

The rheological behaviour of the ink also plays a decisive role in our ability to print 3D complex geometries with tuneable mechanical response or smooth substrates for functional piezoresistive and conductive elements. To print 3D structures with overhangs and complex patterns, the ink should display an yield stress that is high enough to prevent the shape-distorting effect of capillary forces^51^. This requirement is fulfilled by silicone inks containing 12.5% (w/w) modified CNCs. With a yield stress of 1.3 kPa, this ink enables the deposition of distortion-free filaments that make it possible to fabricate parts which extend in 3D, such as the grid-type structures shown in Fig. 2d. While 3D geometries need viscoelastic inks, the smoother regions of the insole required to host piezoresistive and conductive elements can only be formed using inks that are sufficiently fluid to be flattened into a smoother surface through the action of gravity and capillary forces. To satisfy this condition, we opted for inks with a lower CNC concentration of 5.0% (w/w). Optical microscopy analysis of printed samples reveals that this ink leads to surface roughness of 4.2 ± 1.5 µm, which is at least 8 times lower than that achieved with a formulation containing 12.5% (w/w) CNCs (Fig. 2f). Printing experiments show that the smoothness of the substrate is crucial to generate robust electric connectors and piezoresistive elements (Figure S1). Moreover, we found that the introduction of rigid bumps on top of the printed piezoresistive layer improved the sensitivity of the sensor by enhancing force transfer to the sensing element (Fig. 2g). As the presence of rigid bumps may reduce the ergonomics and support of the foot, an additional topological layer can be printed onto the insole to prevent any influence of the bumps on the gait and to ensure the long-term reliability of the final wearable device. Inks containing 12.5% (w/w) CNCs were used for the production of the rigid bumps on top of the sensing layer. Importantly, our results show that the use of modified CNCs and tuneable grid designs allows us to tune the ink rheology and the mechanical properties of the printed material without changing the chemical composition of the base silicone matrix.

Piezoresistive elements were successfully printed on smooth silicone substrates using the functional inks filled with carbon black particles (Fig. 2g). To achieve a piezoresistive response, these inks were diluted with a solvent that induces the formation of a stress-sensitive network of carbon particles upon drying. The rheological behaviour of the piezoresistive ink was optimized for DIW by incorporating fumed silica into the formulation (Figure S2). To complete the set of inks required to print the electronic insoles, we selected a commercially available silver-based formulation. Such ink was used to generate the circuitry of 50–100 µm-thick lines connecting the sensing elements (Fig. 2g). To improve the adhesion of this ink onto the silicone substrate, we perform a pulsed arc plasma treatment on the elastomer using a custom-mounted atmospheric plasma system. This system is directly mounted on the printer to allow for inline surface treatment of the substrate during the fabrication process. The positive effects of such treatment on the adhesion of the silver connectors were confirmed via a tape adhesion test (Figure S3). The sensing capability of the carbon black-silicone composite was evaluated by measuring the change in electrical resistance of the printed piezoresistive element as a function of the applied pressure. Using different ink formulations, we observed that the sensitivity of the piezoresistive elements can be increased by a factor of 3 by tuning the carbon black concentration within the range of 4.0–5.0% (w/w) (Fig. 2h). Further optimization of the type and amount of solvent added to the ink enabled an additional improvement in the stability of the sensors (Figure S4). For the targeted application, a concentration of 4.0% (w/w) carbon black and 80% 1-pentanol was found to be optimal in terms of ink printability and piezoresistive performance. The compression modulus of the material printed from this optimal ink was measured (Figure S2d) and used for the determination of the gauge factor of the printed sensing elements.

### Sensor performance

**Figure 3:**
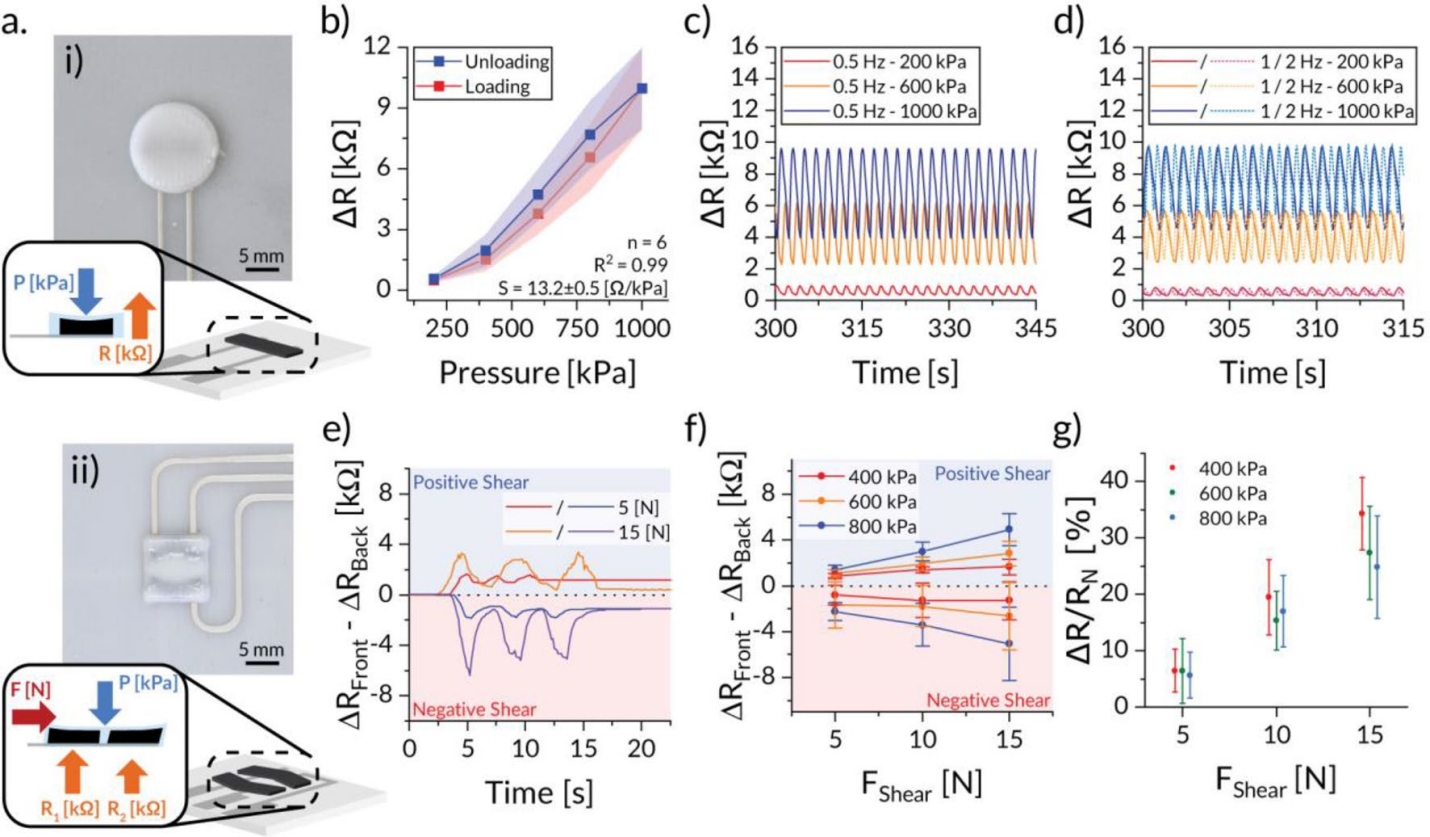
Piezoresistive sensors used to measure normal and shear forces. (**a.i,ii**) Images and schematics of the (**i**) normal and (**ii**) shear force sensors. (**b**–**d**) Responses in the form of change in resistance (∆R) of the normal sensor under (**b**) static, and (**c**) low-frequency or (**d**) high-frequency dynamic conditions. (**e**,**f**) Responses of the shear sensor in the form of the difference in resistance between front and back sensor as a function of (**e**) time and (**f**) applied shear force. (**g**) Sensitivity of the shear force sensor quantified in terms of normalized resistance change (%) under distinct normal and shear forces.

Using the above-described materials and printing system, we developed normal and shear force sensor designs that could be integrated into a flexible insole to monitor gait. Sensors with simple strain gauge design were fabricated by DIW printing a piezoresistive ink with 4.0% (w/w) carbon black on top of a substrate printed with a structural ink containing 5.0% (w/w) CNCs. Normal pressure sensing was achieved using a sensor configuration that employs a single rectangular piezoresistive element sitting on two parallel silver electrodes (Fig. 3a.i). The working principle of this sensor relies on the increase in electrical resistance in the longitudinal conductive path of the sensing gauge induced by the tensile strain due to normal compression^52^. To measure shear forces, we used a second sensor configuration consisting of two piezoresistive elements placed in parallel. In this design, the difference in electrical resistance between the two elements is used to quantify the shear forces applied (Fig. 3a.ii). Piezoresistive elements with a chevron shape were utilized to introduce mechanical compliance in one direction and resistance in the opposite, resulting in strain difference between the two elements under the same shear force. The strain gauges of all sensors were covered with a bump, printed from a more rigid structural ink (12.5% (w/w) CNC), to allow for a more effective force transfer and consistent compression pressure. To show the effectiveness of our 3D printing approach, multiple sensors were manufactured and tested using a set of predetermined testing conditions.

Responses of our normal force sensors were determined by applying load cases resembling human use equal to plantar pressures exerted during walking, running, and other sports activities^53,54^. The performance of the normal sensor to static loads was determined by measuring the change in resistance (**∆R)** 30 s after full loading for several sensors (n = 6) over a pressure range of 200–1000 kPa (Fig. 3b). The average static sensitivity of the sensors was found to be 13.2 ± 0.5 Ω/kPa or 0.22 ± 0.03%/kPa (*R*^2^ = 0.998) for this pressure range. The observed deviations in the peak response values can be attributed to a slight difference in baseline resistance (**R**_**0**_) between sensors, which had a value of 6.1 ± 1.4 kΩ (Figure S5). By calibrating for this baseline offset, the sensors can accurately sense plantar pressures for both low and high impact activities. The static responses were found to be stable over time after full load application in both the loading and unloading cases (Figure S6). From the experimentally measured resistance change of 8.2 ± 1.1 kΩ and the compression modulus of 9.2 ± 0.1 MPa (Figure S2d), we calculated the gauge factor (GF) of the normal force sensors to be 31.2 ± 0.1.

In addition to static loading conditions, the dynamic response of the normal force sensors was assessed as well using selected pressures of 200, 600, and 1000 kPa. Measurements were performed at the frequencies of 0.5 Hz, 1 Hz and 2 Hz to represent slow walking speeds of less than 3 km/h (< 1 Hz) and running speeds faster than 18 km/h (Fig. 3c,d, Figure S7)^55^. After a short stabilization period, the response of the sensor can be seen as a harmonic wave with maxima and minima corresponding to loaded (peak) and unloaded states (valley). The difference between these two values is defined as the dynamic amplitude. These amplitudes were found to be stable over time, with less than 2% peak drift for the time interval between 5 and 30 min (Figure S7). To quantify the sensitivity of the piezoresistive element under these dynamic conditions, we averaged the peak and valley values measured every 5 min and analysed for each test condition (Figure S8). The results indicate that the relative peak response increases linearly with the actuation pressure, leading to a sensitivity of 16.9 ± 0.8% per 100 kPa (*R*^2^ = 0.999). This sensitivity is slightly lower than that measured for the static test, probably due to the transient nature of the applied load. The relative valley values follow the same trend, albeit with a lower variation of 10.1 ± 2% per 100 kPa (*R*^2^ = 0.989). The lower sensitivity obtained for the valley values might be related to the viscoelastic properties of the piezoresistive material, which prevents it from fully returning to its initial state. Notably, the loading frequency did not significantly influence either the peak or valley sensitivities of the piezoresistive sensor (Figure S8b).

The shear force sensors were evaluated by clamping the sensors with normal pressures of 400, 600 and 800 kPa and shearing them in either the negative or positive direction up to a force of 15 N. The positive direction corresponds to a shear force applied towards the toes, while the negative direction is associated with forces pointing to the heel (Fig. 3e). Before shearing, the sensors exhibited an average baseline resistance under clamping pressure (**R**_**N**_) of 7.0 ± 0.8 kΩ. The response of the sensor to the different applied shear forces was quantified by measuring the change in resistance (**R − R**_**N**_) of the piezoresistive elements for clamping pressures of 400, 600 and 800 kPa. We call the change in resistance in the front and the back piezoresistive elements **∆R**_**Front**_ and **∆R**_**Back**_, respectively. The experimental data reveal that, by measuring the difference in resistance change between the front and back piezoresistive elements (**∆R**_**Front**_–**∆R**_**Back**_**)**, the sensors can be used to effectively distinguish the direction of the applied shear force independent of the normal load (Fig. 3f). For normal loads of 600 and 800 kPa, we observe a direct correlation between the measured differential value and the applied shear forces. This correlation allows the detection of the shear force range if the load pressures applied are sufficiently high. Such feature is especially useful for detecting intense activities with high transient shear pressures, such as cutting or jumping. Even though the absolute differential is higher at increased normal pressure, the highest sensitivity, defined as (**∆R/∆R**_**N**_), was achieved at a pressure of 400 kPa (Fig. 3g) with a value of 2.96 ± 0.11%/N (*R*^2^ = 0.99), as opposed to 2.31 ± 0.28%/N for 600–800 kPa. These sensitivities were found to be independent of the shearing direction. The presented results indicate that the developed fully 3D printed piezoresistive sensors are suitable for detecting a wide range of repetitive motions at a variety of speeds and pressures relevant for gait monitoring. For the detection of gait pressures, only the peak response needs to be monitored, which was found to be linear and repeatable for the normal force sensors. In addition to maximum pressure, the intensity of the physical activity can be determined with the dynamic amplitude of the signal, which has a slight error of a few percent on the measured pressure values. The ability to measure the direction and magnitude of shear forces is a unique feature of the developed piezoresistive sensors, complementing the information provided by the normal pressure data. Lastly, a small amount of drift over time was observed during the dynamic tests which was strongest at a slower walking pace. This dependency on frequency and drift could be corrected for by developing an appropriate signal processing algorithm.

### Shoe insole demonstrator

**Figure 4:**
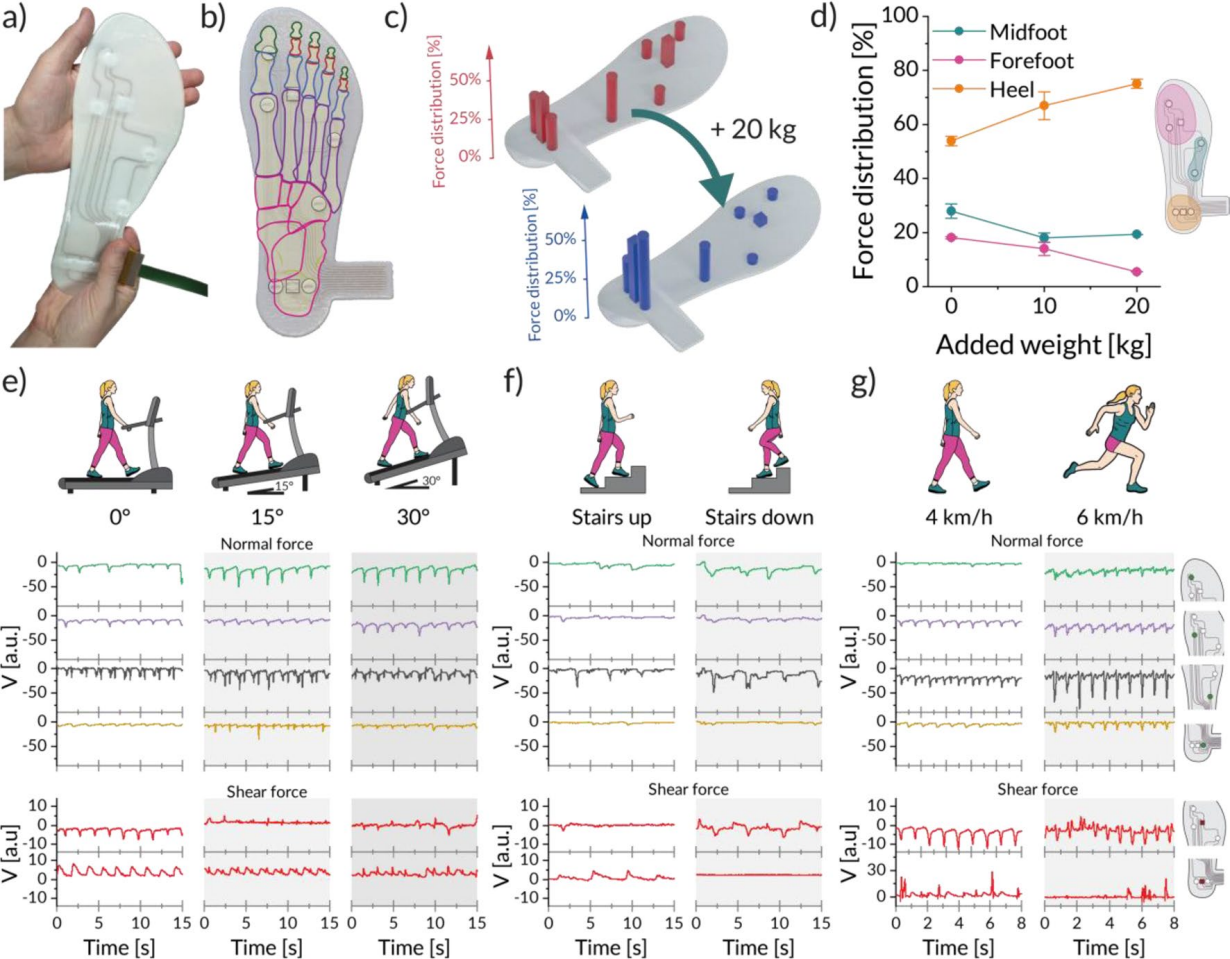
Fully 3D printed insole with embedded sensors and its response for various types of physical activities. (**a**) Photograph of the full insole. (**b**) Overlay of the skeletal structure of a foot over the sensor layout. Squares indicate the shear sensors and circles the normal pressure sensors. (**c**) Normal plantar pressures and shear forces sensed with the embedded sensors over the entire foot for the test subject, without any extra load and with 20 kg of added weight. (**d**) Weight distribution within the insole with increasing added load. (**e**–**g**) Sensor signals and their changes for several activities including (**e**) walking on inclines at 2 km/h, (**f**) walking up and down stairs, (**g**) walking at 4 km/h and jogging at 6 km/h. Illustrations in panels (**e**–**g**) provided by Estevam Quintino (CC BY 4.0).

Using the developed sensors, we demonstrate for the first time a fully integrated 3D printed footwear with both normal and shear force sensing capabilities for real-time gait monitoring (Fig. 4a). The sensor layout of the developed insole was based on the skeletal structure of the foot, with sensors positioned at regions of interest expected to undergo extensive mechanical load (Fig. 4b). The insole was printed using the structural inks (5.0% and 12.5% w/w CNC) to generate the three-dimensional base shape, the piezoresistive ink (4% w/w carbon black) for the sensing elements and the conductive ink for the electrodes and connecting elements. To protect the sensors and electrodes from wear and tear, a monolithic encapsulation layer was also printed on top of the insole base using the structural ink with 5.0% (w/w) CNC. This additional layer resulted in a stiffening effect and an increased sensor response during static compression that enhanced the piezoresistive element sensitivity up to 16.8 ± 1.5 Ω/kPa or 0.3 ± 0.0%/kPa. Furthermore, cross-talk which could be caused by the encapsulation was analysed by re-evaluating the sensors post-encapsulation (Figure S10). The found cross-talk was of an error of less than 0.4% or less than 0.1 kPa, which does not significantly influence the sensor readout.

To evaluate the gait monitoring capabilities of the insole, static and dynamic tests were performed by a test subject of around 70 kg. For these monitoring experiments, the test subject performed several physical activities while wearing the insole. Normal and shear forces were captured during these activities using the integrated sensors. A static evaluation of the normal plantar pressure distribution could also be made, which shows that the highest pressure occurs in the rearfoot. Our results indicate that 57.1 ± 1.7% of the total weight was recorded at the back of the foot, as opposed to 42.8 ± 2.6% in the forefoot (Fig. 4c). These values agree with measurements made using an external plantar pressure measurement device^56^. The pressure detection was further evaluated with the test subject carrying a backpack with 10 and 20 kg of additional weight. This altered both the normal plantar distribution and their stance, with the additional weight shifting the pressure distribution towards the heel (Fig. 4d).

To complement the static analysis, the performance of the shoe insole was further investigated under several dynamic gait modes, including walking in terrains of distinct slopes, stepping up and down a staircase, and running at different speeds (Fig. 4e–g). The results are all reported in terms of a digital voltage output from the normal and shear force sensors. To demonstrate the influence of surface steepness on gait, normal and shear forces were measured during walking on an inclined treadmill. Walking tests were performed at a relaxed pace of 2 km/h on a flat surface, a medium slope of 15°, and a steep slope of 30° (Fig. 4e).

The dynamic sensing data obtained reveal that the test subject compensated for the increase in incline by changing the stance. This is evidenced by a redistribution of the normal pressure, with more weight transferred to the forefoot, which matches previous results measured with an external device^57^. For sensors that did not experience high pressure loads already on flat surfaces, their response did not significantly change under inclined conditions. The change in stance is also captured by the shear force data. These forces are evaluated in terms of differential signals created by subtracting the responses from a pair of gauges. Two sets of gauge pairs are considered, one positioned in the rearfoot and another in the forefoot. For both sets of sensors, when the shear force is applied towards the heels (toes), the differential signal is positive (negative). On a flat surface, our measurements reveal that both shear force signals show a comparable magnitude of around 6.3 ± 0.9 a.u. but of opposite polarities. This means that shear forces are applied in the direction of the toes for the forefoot and in the direction of the heel for the rearfoot, similar to shear forces measured using an insole equipped with optical sensors^58^. When the surface is inclined to 15°, however, the polarity of the voltage in the forefoot changes from negative to positive, which indicates that the shear forces at the fore- and rearfoot are now both applied towards the heel. The amplitude of shear forces detected in the heel decreases by about 18% at a 15° incline and becomes irregular and bidirectional at a 30° incline. This is likely due to the reduction in the step length and change in the cadence with increasing inclination^59^.

Our insole was also tested while stepping up and down a staircase, which constitutes a unique type of gait (Fig. 4f). When the test subject is walking up the stairs, high normal pressures are exerted on all regions of the foot except for the rearfoot, as the whole body needs to be lifted to place the other leg on to the next step. The signal in the rear of the foot is dominated by shear forces, as the heel strikes the next step first before the rest of the foot touches it. A similar scenario is found when the subject is walking down the stairs. Again, high normal pressures are detected in all regions except for the rear of the foot since the subject lands with full weight on the front of the foot before stabilising. This instability results in a bidirectional shear motion similar to that observed with the subject walking at an incline of 30°. In this case, though, high shear forces develop only at the forefoot, which is the region that first touches the next stair step when walking downwards.

Lastly, we tested the response of the insole under faster gaits by having the test subject walk at a speed of 4 km/h and jog lightly at 6 km/h (Fig. 4g). Under these conditions, all sensors were able to record the development of the gait (Figure S10). By increasing the speed from 4 to 6 km/h, we observe a 2.5 ± 0.6 times increase in normal pressure, which agrees with values found in the literature^57,60^. Furthermore, at faster speeds the foot placement becomes less stable, as indicated by shear forces occurring in both directions. These shear patterns are similar to those for inclines at 30°, and walking down stairs. Overall, our real-time gait monitoring tests demonstrate that the combination of normal and shear force sensors, distributed at specific locations across the insole, provide extensive motion data that can be effectively used as blueprints to identify the gait mode and the intensity of the physical activity.

## Conclusion

In summary, we have developed functional inks for the manufacturing of a fully 3D printed insole with integrated piezoresistive sensors that is suitable for the normal and shear pressure monitoring of human gait. By placing these sensors at patient-specific positions of the insole, it is possible to identify and quantify gait in real-world conditions. As the feedstock materials are readily available and can be processed at room temperature using a desktop extrusion-based printer, we expect this manufacturing platform to be cost-effective and to be readily translatable to orthopaedic facilities where the insole can be adapted in-situ to the user by physicians and orthopaedic specialists. Moreover, the biocompatible nature of the silicone used in the ink formulations makes our materials skin‐safe, flexible, and robust for human wear. The proposed technology should therefore open the way towards custom smart footwear that can measure the gait for both rehabilitation and sport performance alike.

## Methods

### Utilised materials

Trimethoxymethylsilane (MTMS) and 1-pentanol were purchased from Sigma-Aldrich. Carbon black micropowder, (Ketjenblack EC-300J) was obtained from Nouryon. Hydrophobic fumed silica (HDK 30) was obtained from Wacker Chemie AG, whereas the cellulose nanocrystals (CNC, CelluForce NCV10) were obtained from CelluForce. The silicone elastomer SYLGARD 184 used throughout the project was supplied by Dow Chemical, while the silver-based ink (Ag Paste 520 EI) and thinner were purchased from Chimet S.p.A.

### Silanization of CNCs

The silanisation of CNCs with MTMS was performed according to an established protocol^61^. Briefly, 1.7 g of MTMS were added dropwise to 500 ml of distilled water (MilliQ) and the pH of the resulting solution was adjusted to pH 4 using HCl. Meanwhile, 5 g of CNCs were dispersed in 250 ml of MilliQ water and the pH of the obtained suspension was adjusted to 4 using HCl. After the pH of the two batches was stabilized, the MTMS solution was added dropwise to the CNC suspension under stirring and the mixture was left for 1 h to enable the silanisation reaction. The suspension was then frozen with liquid nitrogen and freeze‐dried to obtain a linty CNC-MTMS powder.

### Preparation of the inks

The conductive silver paste (Chimet, Ag Paste 520 EI) was used to print the electrodes and the connections of the sensors after dilution with 10% (w/w) thinner. To prepare the piezoresistive silicone-based ink with 4% (w/w) carbon black, 0.20 g of carbon black micropowder was mixed into 3.49 g of 1-pentanol using a planetary mixer (Thinky, ARE-250) for 5 min at 2000 rpm. Later, 0.44 g of hydrophobic fumed silica and 3.96 g of SYLGARD 184 Elastomer Base were added to the batch and mixed again in the planetary mixer for 5 min at 2000 rpm. The obtained paste was then milled down to 10 μm using a 3-roll-mill (EXAKT Technologies, EXAKT 80). Finally, SYLGARD 184 Curing Agent was added to the mixture in a 1:10 (w/w) ratio with respect to the amount of Elastomer Base present in the paste after milling. The piezoresistive ink was then directly used to print the sensing elements. To prepare the structural silicone-based ink with 5% (w/w) modified CNC, we mixed 0.50 g of MTMS-coated CNC into 8.64 g of SYLGARD 184 Elastomer Base using the planetary mixer for 5 min at 2000 rpm. After milling the obtained paste down to 10 μm using the 3‐roll‐mill, we added 0.86 g of SYLGARD 184 Curing Agent to the mixture. The structural ink with 12.5% (w/w) MTMS-coated CNC was prepared following the same procedure. In this case, 1.25 g of MTMS-coated CNC were added into 7.95 g of SYLGARD 184 Elastomer Base, with 0.80 g of SYLGARD 184 Curing Agent being added at the end of the process.

### Printing system

The printing system used throughout the study was assembled in‐house based on a Stepcraft D420 rig (Figure S12a). Such rig was fitted with a custom-made printing head able to hold 3 tools at once (Figure S12b). The structural silicone-based inks were printed using a progressive cavity pump (Preeflow, eco-PEN300), whereas the other ink formulations were deposited using an air pressure controller. The rig was also fitted with a plasma system (Relyon, plasmabrush PB3) to activate the inks and improve interlayer adhesion. As the printed structures are thin and lightweight, a heating plate was built into the printer to ensure thermal curing. In case taller geometries need to be printed using our system, an additional heating chamber will have to be used to make sure the prints are properly cured. All G-codes used for printing were obtained using a custom-written slicer developed in Grasshopper for Rhinoceros (McNeel).

### Mechanical testing and rheology

The compression and tensile tests were all performed using a tabletop mechanical testing machine (AGS-X, Shimadzu). Both compression and tensile measurements were performed at a displacement rate of 5 mm/s. For compression tests, we used cylindrical samples with 6 mm thickness and 19 mm in diameter. For tensile tests, dogbone specimens were employed. All rheological tests were carried out at 25 °C on a stress-controlled compact rheometer (Anton Paar MCR 302) using a sandblasted parallel plate geometry (PP25) with a 1 mm gap. Amplitude sweeps were performed at a frequency of 1 Hz. Elastic recovery tests were conducted by alternating oscillatory measurements at 1% strain and 1 Hz, and rotational measurements at a shear rate of 50 s^−1^, to simulate the forces applied to the inks during the printing process.

### Adhesion tests

Adhesion tests were carried out on 1 × 1 cm^2^ silver patches obtained by printing the conductive ink on different substrates. Each square was cut in a grid with a scalpel, resulting in 49 different square regions. Scotch Shipping Packaging Tape was applied to each sample and removed after 10 s adhesion time. The adhesion score was then evaluated according to the ASTM F1842-15 standard (Figure S3)^62^.

### Sensor characterization

The static and dynamic responses of all sensors were quantified by measuring the electrical resistance of the piezoresistive element while applying compressive pressures using a mechanical tester (Instron 3340). The resistance of the sensors was measured using an Agilent 34410/11A Digital Multimeter in two-point probe mode. Viscoelastic effects were reduced by warming up the sensors through cycling them to the maximum applied load (1000 or 800 kPa) at a cycling speed of 20 kPa/s.

The evaluation of the sensors under static loads was carried out at pressures between 200 and 1000 kPa at steps of 200 kPa. Measurements were taken for both loading and unloading cycles. Response of the sensors were processed using a Python script. The electrical resistance of the piezoresistive element was measured 30 s after application of the maximum load to allow for signal stabilisation.

The hysteresis tests (Figure S9) were carried out by applying loads of 200, 600 and 1000 kPa at a load rate of 20 kPa/s in a total of 3 cycles. The data obtained were processed using a Python script to determine the percentage of hysteresis.

The dynamic response of the sensors was assessed at compressive pressures of 200, 600, and 1000 kPa using a dynamic mechanical tester (Bose Electroforce 3400) at cycling speeds of 0.5, 1 and 2 Hz. The signals were processed using a Python script to determine the time-dependent characteristics of the electrical output.

Shear tests were carried out in a mechanical tester (Instron 3340) under compressive loads of 400, 600, and 800 kPa. Shear forces of 5, 10, 15, 20 N were applied successively and separately for both sensing directions using a custom-built shearing setup. Shear force loads were recorded using a dedicated load cell (Futek FSH00096) and controller (Futek IPM650).

### Insole characterization

For the insole tests, the sensor signals were captured using a custom-made voltage divider interfaced with a microcontroller (Adafruit Feather 32u4). Data were collected using an in‐house developed Python suite that could capture and process the data.

To be able to connect the electronics readout circuit to the soft insole, silver paste (Chimet, Ag Paste 520 EI) was stencil-printed on top of the leads from the sensors to create a connection with a flexible copper patterned printed circuit board (PCB). After curing, the connections were encapsulated with room-temperature vulcanizing (RTV) silicone to create a robust contact.

### Ethical approval

Tests on the printed insole were performed with the assistance of a human participant. Informed consent was obtained from the participant prior to the tests. The use of human participants in the project was approved by the École Polytechnique Fédérale de Lausanne Human Research Ethics Committee (HREC), No: 016-2021. All tests were carried out in accordance with relevant guidelines and regulations.

## Supplementary Information

Supplementary Information 1.

Supplementary Information 2.

Supplementary Information 3.

## Data Availability

Supplementary information is attached in the form of a .pdf document with several additional plots and graphics, and two videos. The data supporting the findings of this study are available from the corresponding authors upon reasonable request.
