# Supplementary material for: Digital manufacturing of personalised footwear with embedded sensors
Source: Sci Rep. 2023 Feb 3;13:1962. doi: 10.1038/s41598-023-29261-0 (PMC9898262; doi:10.1038/s41598-023-29261-0)
Supplement: Supplementary file 1 — Supplementary Information 1. [file 41598_2023_29261_MOESM1_ESM.docx]

SUPPLEMENTARY INFORMATION TO:

Digital Manufacturing of Personalised Footwear with Embedded Sensors

Marco R. Binelli^1*^, Ryan van Dommelen^2*^, Yannick Nagel^3^, Jaemin Kim^2^, Rubaiyet I. Haque^2^, Fergal B. Coulter^1^, Gilberto Siqueira^3^, André R. Studart^1^ and Danick Briand^2^

^1^ Complex Materials, Department of Materials, ETH Zürich, 8093 Zürich, Switzerland

^2^ Soft Transducers Laboratory, EPFL Lausanne, 2000 Neuchâtel, Switzerland

^3^ Applied Wood Materials Laboratory, EMPA Dübendorf, 8600 Dübendorf, Switzerland

**Figure S1.** Surface roughness of tracks printed on the substrates printed from different concentrations of CNC-reinforced ink.

**Figure S2.** Rheological and mechanical characterization of the carbon black-fumed silica-Sylgard 184 composite ink used throughout the study. a) Amplitude sweep of the ink run immediately after preparation at 1 % deformation. b) Flow curve at 25 ˚C. c) Simulation of material recovery after printing. Briefly, oscillatory measurements at 1 % deformation and 1 Hz frequency are performed on uncured ink after rotational runs performed at 50 s^-1^. The almost instantaneous recovery of G’ and G” after the intense shearing demonstrates the ability of the ink to retain its mechanical stability even after the intense shear experienced during the extrusion through the nozzle. d) Compression testing of cast cylindrical samples of the piezoresistive ink at 5 mm/min.

**Figure S3.** Results of the peel test on the adhesion of printed silver electrodes after different surface treatments on the silicone substrates.

**Figure S4.** Effect of solvent used and carbon black concentration on the resistivity (a) and on the sensitivity (b) of the printed piezoresistive elements. For 'Pentanol-extracted' samples we refer to samples printed after all solvent has been evaporated in a vacuum planetary mixer.

**Figure S5.** Individual normal sensor responses as a function of static pressure loads for multiple sensors.

**Figure S6.** Sensor response for compressive pressures ranging from 200 and 1000 kPa applied for 90 seconds.

**Figure S7.** Dynamic tests at 200, 600 and 1000 kPa for a) 900 cycles (0.5 Hz), b) 1800 cycles (1 Hz), and c) 3600 cycles (2 Hz).

**Figure S8.** Change of peak and valley response over a) increased pressure, and b) increased speed.

**Figure S9.** Hysteresis loops for unencapsulated sensors.


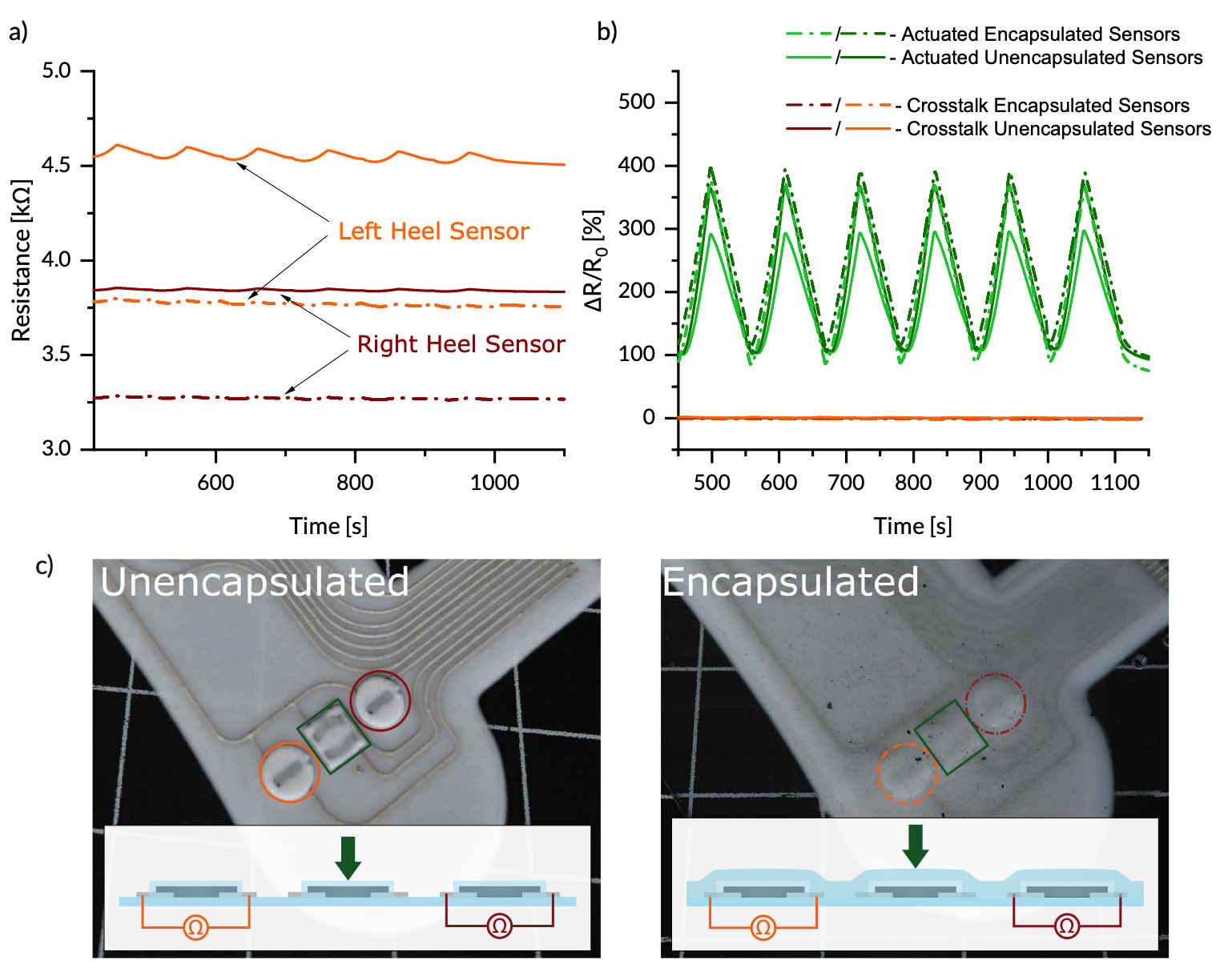


**Figure S10.** Cross-talk test on two sensors tested before and after encapsulation. Data recorded a) before encapsulation of the sensors and b) after encapsulation. c) Photograph and measurement methods showing actuated and recorded sensors for both encapsulation states.

**Figure S11.** Extended recorded data of the in-sole being used during slow walking (2 km/h), walking (4 km/h), and light jogging (6 km/h) activities.


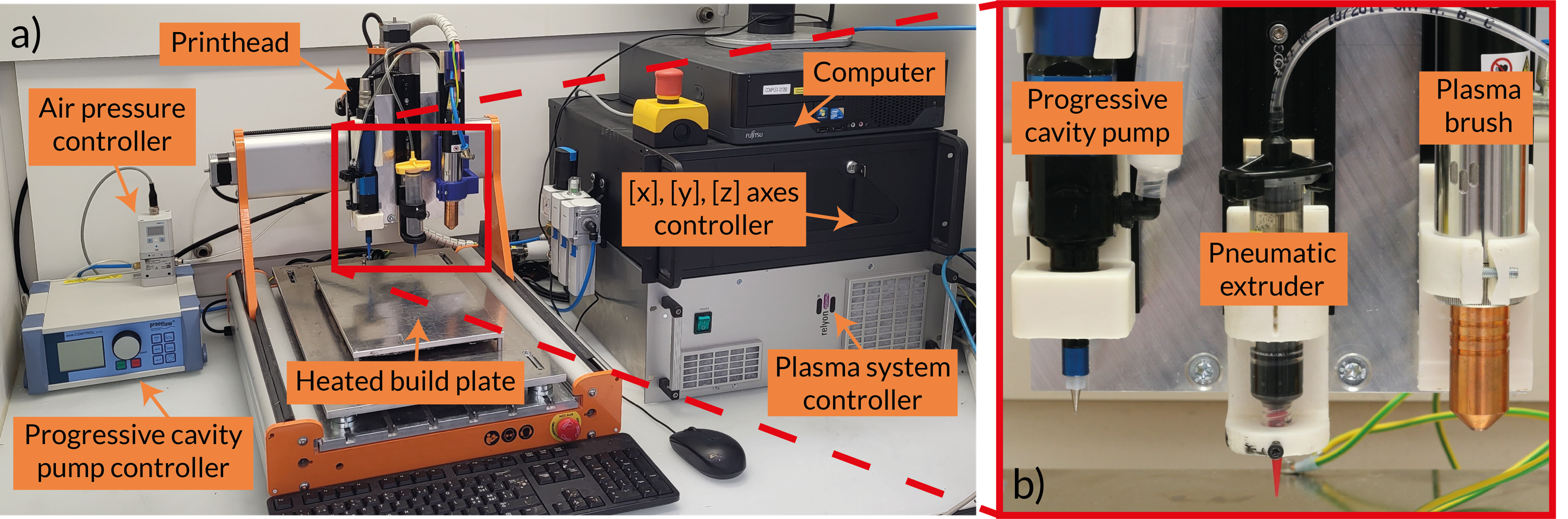


**Figure S12.** Photograph of the printhead used. a) Image of the overall printing setup. b) Close-up view of the printhead with the tools used. From left to right, the progressive cavity pump for the structural ink, the pneumatic extrusion cartridge containing the piezoresistive ink, and the plasma brush used for activating the silicones.
