# Supplementary material for: Digital manufacturing of personalised footwear with embedded sensors
Source: Sci Rep. 2023 Feb 3;13:1962. doi: 10.1038/s41598-023-29261-0 (PMC9898262; doi:10.1038/s41598-023-29261-0)
Supplement: Supplementary file 2 — Supplementary Information 2. [file 41598_2023_29261_MOESM2_ESM.docx]

**Supporting Video 1)** Time-lapse video of the printing process of an insole, in particular of the plasma treatment, the printing of silver connectors and the printing of piezoresistive elements.

**Supporting Video 2)** Manipulation of a finished printed device, demonstrating the stability and the flexibility of the fabricated insoles.
